# Estimating detection for salt-marsh songbirds during winter using the double-pass rope-drag technique

**DOI:** 10.1371/journal.pone.0281535

**Published:** 2023-02-13

**Authors:** Bryan D. Watts, Fletcher M. Smith, Chance H. Hines

**Affiliations:** 1 Center for Conservation Biology, College of William and Mary, Williamsburg, VA, United States of America; 2 Wildlife Resources Division, Georgia Department of Natural Resources, Non-Game Conservation Section, Brunswick, Georgie, United States of America; Texas State University, UNITED STATES

## Abstract

Bird species that are restricted to tidal marshes during one or all of their life stages are under increasing pressure from sea-level rise. To date, most of the research focused on this group has been conducted during the breeding season despite the fact that more than half of the annual cycle is spent on wintering grounds and the high likelihood that the winter period is the most critical time for adult survival. We used a double-pass rope-drag technique to estimate the winter abundance of sharp-tailed sparrows (*Ammospiza nelson* and *A*. *caudacutus* collectively), seaside sparrows (*A*. *maritimus*) and marsh wrens (*Cistothorus palustris*) within tidal marshes of Virginia along 102 60X250 m transects between January and March, 2014. We used the first pass to remove birds from the transect and the second pass was used to estimate detection probabilities. The technique was highly effective producing detection rates of 98% for sharp-tailed sparrows, 95% for seaside sparrows, and 91% for marsh wrens. We conducted three rounds of surveys and found that species-specific detection rates were comparable when we restricted our analyses to two survey rounds. Availability and abundance estimates deviated to a greater degree than detection rates when restricting data to that collected during only two rounds but confidence intervals overlapped for all three taxa, regardless of which two survey periods were used for the comparison. However, results were less precise when we restricted our analyses to two of three rounds with confidence intervals averaging 13%, 45%, and 14% larger for detection, availability, and abundance respectively. The double-pass rope-drag technique provides an effective, unbiased sampling technique to estimate winter songbird abundance in saltmarsh habitat provided that at least two rounds are used and increasing the number of survey rounds will result in more precise estimates.

## Introduction

Bird species that specialize on tidal marshes for one or all of their life stages are of increasingly high conservation concern due to the impacts of rising seas on this habitat type [1, 2]. Several species of New World sparrows are adapted to tidal marshes [3, 4] including three *Ammospiza* species that are complete marsh obligates. The Saltmarsh Sparrow (*Ammospiza caudacutus*) is dependent on tidal marshes throughout its entire life cycle and appears to face a demographic crisis on the breeding grounds related to a decrease in nest survival due to increased frequency of tidal flooding [1, 5, 6] that some authors suggest may pose a risk of extinction as sea-level rise progresses over the next several decades [2, 7]. The Seaside Sparrow (*A*. *maritimus*) is also restricted to tidal marshes throughout its entire life cycle and is the dominant breeding sparrow in tidal marshes along much of the Atlantic and Gulf Coasts [8]. The Nelson’s Sparrow (*A*. *nelsonii*) includes two inland-breeding subspecies (inland; *A*. *n*. *nelsoni*, James Bay; *A*. *n*. *alter*) that depend on non-tidal wetlands and a coastal subspecies (Acadian; *A*. *n*. *subvirgatus*) that nests predominantly in tidal salt marshes [9] along the Northeast Coast. Marsh wrens (*Cistothorus palustris*) occur in freshwater and tidal marshes throughout North America during their entire life cycle [10]. All four of these species converge on the South Atlantic and Gulf Coasts of North America for the winter where they spend more than half of their annual cycle and are completely dependent on marshes [11–13].

Relatively little research has been conducted on the winter ecology and distribution of these marsh birds [but see 11–15] despite the prominence of this season within their annual cycle and the likelihood that this period is the most critical time for adult survival [15, 16]. One of the challenges in working with this group during the winter is the difficulty of detecting them. *Ammospiza* sparrows are notoriously difficult to detect during the winter period [17]. They forage on the ground and are typically seen only when flushed, they are difficult to flush, they generally do not call when flushed, they fly only short distances back into cover and they do not tend to flock. Availability of an effective survey technique has been an impediment to addressing various aspects of their winter ecology.

Rope dragging is a technique commonly used to survey birds in open habitats such as grasslands and emergent marshes [18, 19]. The technique attempts to flush birds that are concealed in dense vegetation to improve detection rates. The approach has been employed most often to flush incubating birds in order to discover nests [e.g., 20–22] but has also been used to locate and capture nonbreeding birds [e.g., 23–25]. In general, the use of this technique during the nonbreeding period has been to locate birds or to assess relative abundance with little consideration of the detection rates required for an unbiased estimate of density. Our ability to produce an unbiased estimate of density is the key to estimating population size, documenting spatial distribution and assessing temporal trends.

Our goal with this project is to develop a survey technique with a high detection rate that can be used to accurately quantify abundance for species that are typically difficult to detect and to evaluate whether additional survey rounds increase detection. Here, we use a double-pass rope-drag technique where the second pass is used to estimate detection probabilities.

## Methods

### Study area

**Fig 1:**
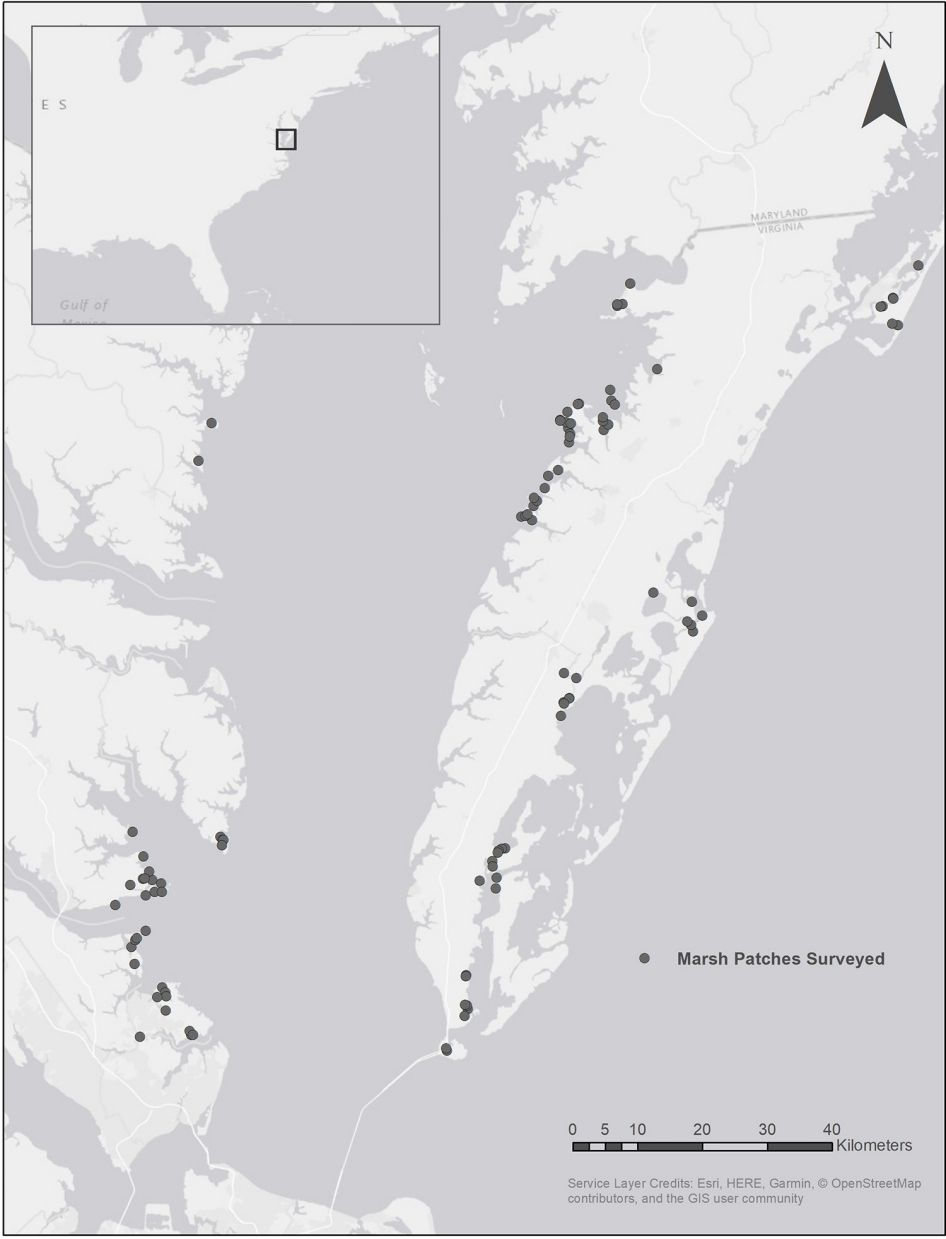
Map of marsh patches surveyed for sharp-tailed sparrows (*Ammospiza nelson* and *A*. *caudacutus* collectively), seaside sparrows (*A*. *maritimus*) and marsh wrens (*Cistothorus palustris*) in Virginia, USA during winter 2014 using the double-pass rope-drag technique. Figure was created using ArcGIS® 10.7.1. Basemap provided by: Esri, HERE, Garmin, © OpenStreetMap contributors, and the GIS user community.

We surveyed sharp-tailed (Nelson’s and saltmarsh sparrows), seaside sparrows, and marsh wrens throughout the outer Coastal Plain of Virginia during the winter of 2014. We worked within 102 patches of salt marsh (defined as marshes exposed to 18–30 ppt salinity) composed of smooth cordgrass (*Sporobolus alterniflorus*), black needlerush (*Juncus roemerianus*), salt meadow hay (*S*. *pumilis*), saltgrass (*Distichlis spicata*), and saltbush (*Baccharis halimifolia*). Field sites were distributed among the Delmarva coastal bay, the Delmarva Chesapeake Bay and the Western Shore Chesapeake Bay (Fig 1). The Delmarva coastal bay includes the seaward margin of the Delmarva Peninsula from the mouth of the Chesapeake Bay to the Maryland-Virginia border. An outer chain of 14 barrier islands protects an extensive lagoon system that contains more than 85,000 ha of tidal marsh, mudflats and open water. The Chesapeake Bay margin of the Delmarva Peninsula includes extensive (>10,000 ha) tidal marshes extending from the Maryland-Virginia border south to Northampton County. The Western Shore of the Chesapeake Bay also includes extensive (>20,000 ha) tidal marshes that occur along the main stem of the Bay and extend up the major tributaries to the location of salinity transition zones.

### Site selection

We screened marsh patches for inclusion in this study based on geographic location, size, access, and distance from other study marshes. The study area supports a large collection of marsh patches. In order to have comparable samples between regions (Delmarva coastal bay, Delmarva bayside, western shore of Chesapeake Bay) of the study area we developed a complete pool of candidate marshes within each region that were large enough to support transect plots. We eliminated patches that were not readily accessible by land or boat or for which we could not secure landowner permission. We considered “independence” as a selection criterion by considering the likelihood of birds moving between patches. Pairs of marsh patches were considered to be “dependent” if they were within the distance focal species are expected to move [26] or if they were not separated by barriers (e.g. waterway, roadway) to movement. For pairs of patches that were likely to be dependent, we randomly selected one for inclusion. Mean nearest-neighbor distance between selected marshes was 759 ± 108 (SE) m which falls beyond the expected seasonal movement distance. We believe that marshes included within the study were representative of marshes within the study area. Final sample sizes were comparable between regions including 37, 33 and 32 patches for Delmarva coastal bay, Delmarva Chesapeake Bay and Western Shore Chesapeake Bay respectively.

### Bird surveys

We used double-pass rope-drag transects to survey sparrows and wrens within marsh sites. Transects were 60m wide and their length varied from 200–250 m depending on the available space and placement within the marsh patch. The 60m rope (0.635 cm diameter) was weighted in the middle by two plastic containers filled with stones to create additional vegetation disturbance. A transect was surveyed at approx. 0.8 m/s^-1^ by three people where two were stationed on either end of the rope and the third walked <5 m behind the center of the rope. The third observer mapped the transect tracks to ensure that the survey was performed in a straight line. Once the entire transect length was surveyed (first pass) the observers immediately reversed direction to pass over the same area a second time (second pass) following the gps track and observer footprints to ensure the exact same path was followed on the return pass. When birds were flushed, we recorded whether the bird landed within or outside the transect area. If birds landed within the transect area ahead of the rope, we monitored their landing location until the rope passed over them again and repeated the process until the bird flushed outside the transect. All birds that landed within the transect after flushing were eventually flushed off of the transect. Mapping allowed observers to distinguish between birds detected on the first pass from new birds detected on the second pass. Flushing birds were recorded by species group. Seaside sparrows and marsh wrens could be readily identified to species. Nelson’s sparrows and saltmarsh sparrows were combined into a single group referred to as “sharp-tailed sparrow” due to the difficulty in distinguishing these two species visually when birds quickly fly to concealing vegetation.

We attempted to survey all transects three times between 12 January and 21 March, 2014, but were unable to complete the third survey at two transects. Mean time between surveys was 8.7 days (SE = ±0.9) and no surveys were conducted on the same date. Surveys were conducted between mid-falling and mid-rising tides to avoid biases produced by marsh inundation during high tides. We learned during previous work in the marshes that the sparrows make local movements to “high ground” including marsh-upland ecotones and shrub patches during high tides making density estimates for marsh patches problematic. Our objective was to estimate density when birds are naturally distributed across the marshes during feeding periods.

### Data analysis

We considered the double-pass rope-drag as a removal experiment, which means that we counted birds that were removed from the transect during the first pass of the rope and then we counted the remaining birds that we flushed during the second pass. This allowed us to estimate detection probabilities for each species using generalized multinomial N-mixture models [27, 28]. We created a single model for each species and models consisted of three hierarchical levels: 1.) detection probability (σ) described the proportion of available individuals we actually observed during surveys and was informed by the removal experiment (two passes), 2.) availability (λ) accounts for temporary immigration and emigration which described the proportion of local individuals currently present in the habitat during surveys and was informed by repeated visits (three double pass surveys), and 3.) abundance (Φ) described the total number of birds that potentially use the transect area.

We first evaluated whether a negative binomial or Poisson distribution best fit our model by examining log-likelihood scores. We did not include covariates and we assumed there was zero mortality, permanent immigration and permanent emigration because our goal was to evaluate the efficacy of the double-pass rope-drag method in determining σ and how additional surveys affect the precision of σ, Φ and λ [29]. To evaluate the utility in surveying three vs two rounds, we compared estimates for σ, λ, and Φ derived from the model using our full data set (three survey rounds) with datasets restricted to the three possible combinations of two survey rounds. All analyses were performed using the R programming language [30] in the package “unmarked” [31].

### Ethics statement

This study was observational with no bird captures or handling required.

## Results

We detected 937 birds during three rounds of rope drag surveys at 100 marshes and two rounds of surveys at two marshes. Mean time for a single pass of the survey transect was 5.5 min (±0.1 SE). The highest counts recorded during a single survey included 13 sharp-tailed sparrows, 6 seaside sparrows, and 4 marsh wrens. We detected the vast majority of birds on the first rope-drag pass including 675 (98.0%) sharp-tailed sparrows, 154 seaside sparrows (95.4%), and 86 (91.5%) marsh wrens.

### Detection

**Fig 2:**
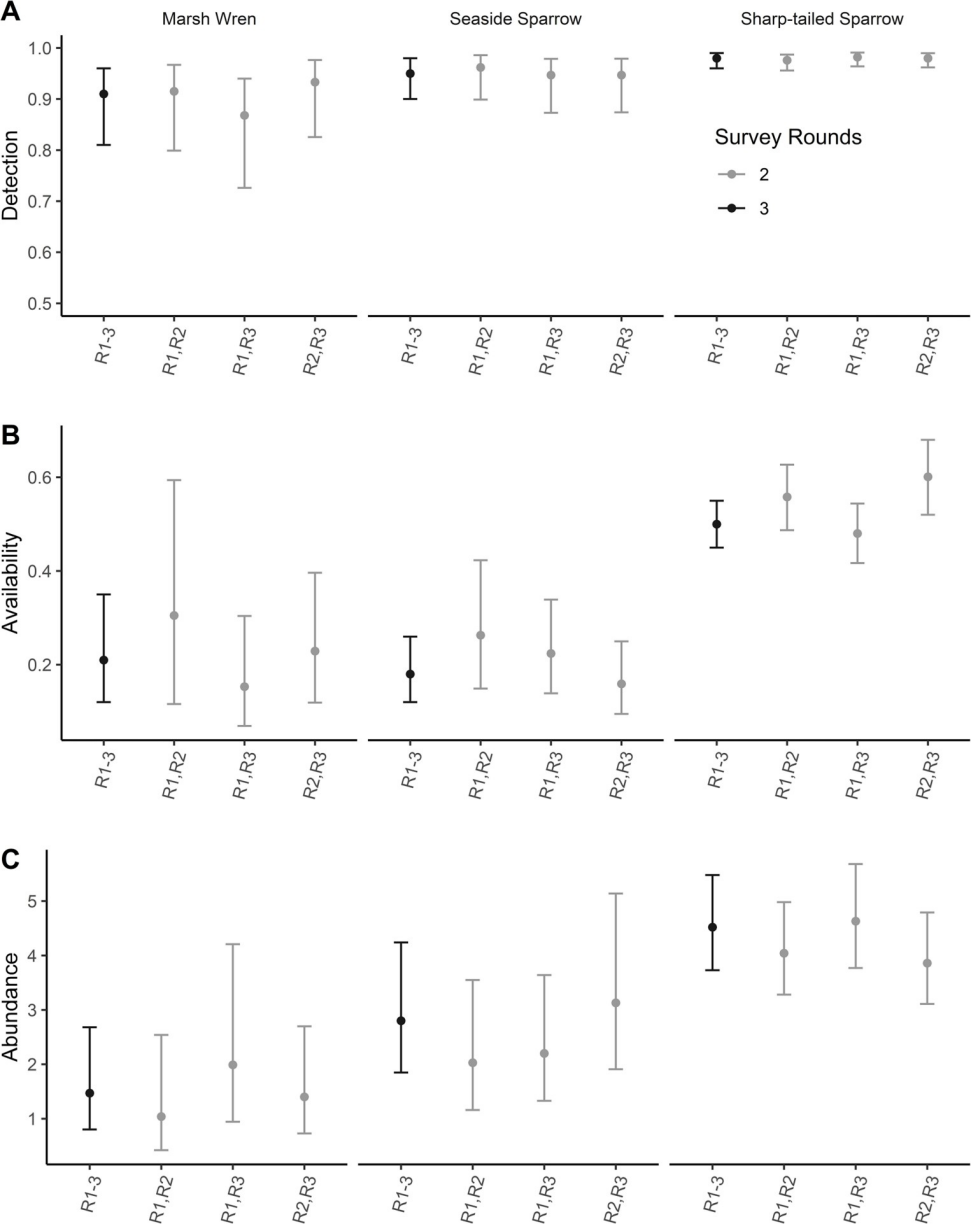
Changes in estimated detection (A), availability (B) and abundance (C) for sharp-tailed sparrows (*Ammospiza nelson* and *A*. *caudacutus* collectively), seaside sparrows (*A*. *maritimus*) and marsh wrens (*Cistothorus palustris*) in Virginia, USA during winter 2014 using the double-pass rope-drag technique with different combinations of survey rounds. R1-3 represents results from analyses using the full data sets Rounds 1–3, R1,R2 represents results from analyses using data restricted to that collected during Rounds 1 and 2, R1,R3 represents results from analyses using data restricted to that collected during Rounds 1 and 3, and R2,R3 represents results from analyses using data restricted to that collected during Rounds 2 and 3.

The negative binomial distribution (Δlog-likelihood = 0.0), better fit our data than the Poisson distribution for sharp-tailed sparrows (Δlog-likelihood = -39.1), seaside sparrows (Δlog-likelihood = -235.8) and marsh wrens (Δlog-likelihood = -9.9). We estimated σ for all survey transects using the full survey dataset (three survey rounds) at 0.98 (0.96:0.99, 95% CI) for sharp-tailed sparrows, 0.95 (0.90:0.98) for seaside sparrows, and 0.91 (0.81:0.96) for marsh wrens. When limiting our dataset to two of three rounds, σ deviated from the full survey dataset by <1% for sharp-tailed sparrows, <1–1% for seaside sparrows, and 1–4% for marsh wrens. Detection rate 95% CI derived from two and three survey rounds were overlapping for all taxa so additional survey effort did not result in significantly different estimates (Fig 2A). However, 95% CI derived from two surveys were less precise, averaging 13% larger than those derived from the full dataset. The difference between parameter estimates derived from two survey rounds compared to three rounds and the range of 95% parameter estimate CI were both greatest for marsh wrens and lowest for sharp-tailed sparrows (Fig 2).

### Availability

We estimated λ for all survey transects using the full survey dataset at 0.50 (0.45:0.55, 95% CI) for sharp-tailed sparrows, 0.18 (0.12:0.26) for seaside sparrows and 0.21 (0.12:0.35) for marsh wrens. When limiting our dataset to two of three rounds, λ estimates deviated from the full survey dataset by 4% - 20% for sharp-tailed sparrows, 12%-45% for seaside sparrows, and 7%-43% for marsh wrens. Confidence intervals for λ estimates derived from one and two survey rounds were overlapping so additional survey effort did not result in significantly different estimates (Fig 2B). However, 95% CI derived from two surveys were less precise, averaging 45% larger than those derived from the full dataset though this differed by species (Fig 2B). Mean difference and the range of 95% CI were both greatest in marsh wrens and lowest in sharp-tailed sparrows (Fig 2).

### Abundance

We estimated Φ for all survey transects using the full survey dataset at 4.52 (3.73:5.48, 95% CI) for sharp-tailed sparrows, 2.8 (1.85:4.24) for seaside sparrows, and 0.91 (0.81:0.96) for marsh wrens. When limiting our dataset to two of three rounds, Φ deviated from the full survey dataset by 2% - 15% for sharp-tailed sparrows, 12%-28% for seaside sparrows, and 5%-35% for marsh wrens. Confidence intervals for Φ derived from one and two survey rounds were overlapping so additional survey effort did not result in significantly different estimates (Fig 2C). However, 95% CI derived from two surveys were less precise, averaging 14% larger than those derived from the full dataset though this differed by species (Fig 2C). Mean difference and the range of 95% CI were both greatest in marsh wrens and lowest in sharp-tailed sparrows (Fig 2).

## Discussion

Assessing the density during the winter period of cryptic birds that naturally occur in low densities has been problematic due to low detection rates, the difficulty of physically sampling enough habitat to overcome low densities, and the lack of a repeatable sampling technique [24, 32, 33]. The double-pass rope-drag method provides an effective sampling technique to estimate abundance that avoids bias associated with high tide marsh inundation. Detection probabilities were very high with 91–98% of birds recorded during the first pass. The second pass does not appear to be critical as survey techniques with detection probabilities near one provide reliable indices that can be used to estimate population growth and decline [34, 35]. However, the second pass requires a relatively small time investment (<6 min on average per pass) and provides a probabilistic assessment of detection rates that is an essential element of a reliable abundance estimate [36]. A second important element of the technique is that it samples a defined space.

Previous work within the southeast United States with marsh sparrows during the winter period has focused on captures to assess species composition [12–14], contaminant exposure [37, 38], or survivorship [15]. These efforts have typically maximized captures by capitalizing on high-tide events that concentrate sparrows within roosts. Although counts within these roosts may provide some index of relative abundance, high-tide roosts draw birds from considerable distances depending on the specifics of the tide and surrounding landscape such that the underlying size of the sampled area needed for density estimation is unknown. Sampling fixed areas during the low-tide period appears to provide a more consistent and useful measure, though capture results within study sites could provide additional demographic information such as age- and sex-class which are key to projecting population stability [39].

The double-pass rope-drag method we employed is intended to be used to derive density estimates by multiplying λ by Φ and the area sampled. We found that, unlike call-playback surveys conducted for some cryptic bird species [40], the addition of a third survey round did not lead to significantly different results for any of the three metrics N-mixture models provide. Detection estimates were similar to each other regardless of how many or which survey rounds we used to estimate the metric, with two-round estimates ranging no more than 5% from those derived from the full three-round dataset. λ and Φ, on the other hand, deviated by as much as 43% and 35%, respectively, for marsh wrens and 45% and 28% for seaside sparrows, which is more or nearly more than twice the greatest variation we found for sharp-tailed sparrows (Fig 2).

We encountered more than four times as many sharp-tailed sparrows as we did seaside or marsh wrens and the greater variation in Φ and λ metrics may be due to differences in sample size. Future studies should take this into account if surveys are intended to estimate parameters for birds in low densities. The taxa considered here are complete marsh obligates during the winter and spend a disproportionate amount of their annual cycle on their winter grounds [41–43]. Because their winter habitat is being subjected to threats from sea-level rise, invasive plant species, eutrophication, and human modification [44–49], it is likely that the winter life stage will play an increasingly pivotal role in their conservation. Documenting distribution during this period serves to inform jurisdictions about their respective roles in conservation planning and efforts. The double-pass rope-drag transect is a sampling technique that may be deployed to investigate density over a range of spatial scales from patches to an entire winter range. The technique may also be used to assess patterns of habitat use or other ecological questions involving spatial distribution. Because the technique is repeatable it is particularly attractive for assessing temporal trends. This study is the first to utilize the technique to assess abundance within saltmarsh habitat.

## References

[1] Bayard TS, Elphick CS. Planning for sea-level rise: Quantifying patterns of saltmarsh sparrow (*Ammodramus caudacutus*) nest flooding under current sea-level conditions. Auk. 2011. 128:393–403.

[2] Correll MD, Wiest WA, Hodgman TP, Shriver WG, Elphick CS, McGill BJ, et al. Predictors of specialist avifaunal decline in coastal marshes. Conserv Biol. 2016. 31:172–182. doi: 10.1111/cobi.12797 27542096

[3] Grenier JL, Greenberg R, Benkman C. A biogeographic pattern in sparrow bill morphology: Parallel adaptation to tidal marshes. Evolution. 2005. 59:1588–1595. doi: 10.1554/04-502 16153044

[4] Greenberg R, Olsen B. Bill size and dimorphism in tidal-marsh sparrows: island-like processes in a continental habitat. Ecol. 2010. 91:2428–2436. 10.1890/09-1136.1 20836464

[5] Shriver WG, Vickery PD, Hodgman TP, Gibbs JP. Flood tides affect breeding ecology of two sympatric sharp-tailed sparrows. Auk. 2007. 124:552–560.

[6] Gjerdrum C, Sullivan-Wiley K, King E, Rubega MA, Elphick CS. Egg and chick fates during tidal flooding of saltmarsh sharp-tailed sparrow nests. Condor. 2008. 110:579–584.

[7] Field CR, Bayard TS, Gjerdrum C, Hill JM, Meiman S, Elphick CS. High-resolution tide projections reveal extinction threshold in response to sea-level rise. Glob Chang Biol. 2016. doi: 10.1111/gcb.13519 27684043

[8] Post W, Greenlaw JS. Seaside sparrow (*Ammodramus maritimus*). In The Birds of North America Online (A. Poole, Editor). Cornell Lab of Ornithology, Ithaca, NY, USA. 2009. http://bna.birds.cornell.edu/bna/species/127

[9] Nocera JJ, Fitzgerald TM, Hanson AR, Milton GR. Differential habitat use by Acadian Nelson’s sharp-tailed sparrows: implications for regional conservation. J Field Ornithol. 2007. 78:50–55.

[10] Kroodsma DE, Verner J. Marsh Wren (*Cistothorus palustris*), version 1.0. In Birds of the World (A. F. Poole, Editor). Cornell Lab of Ornithology, Ithaca, NY, USA. 2020. 10.2173/bow.marwre.01

[11] Greenlaw JS, Woolfenden GE. Wintering Distributions and Migration of Saltmarsh and Nelson’s Sharp-tailed Sparrows. Wilson J Ornithol. 2007. 119:361–377.

[12] Michaelis AK. Winter ecology of sharp-tailed and seaside sparrows in North Carolina. M.S. thesis. 2009. University of North Carolina at Wilmington, Wilmington.

[13] Watts BD, Smith FM. Winter composition of Nelson’s sparrow (*Ammodramus nelsoni*) and saltmarsh sparrow (Ammodramus caudacutus) mixed flocks in coastal Virginia. Wilson J Ornithol. 2015. 127:387–394.

[14] Post W. The status of Nelson’s and Saltmarsh Sharp-tailed sparrows on Waccasassa Bay, Levy County, Florida. Florida Field Naturalist. 1998. 26:1–6.

[15] Winder VL, Michaelis AK, Emslie SD. Winter survivorship and site fidelity of Nelson’s, Saltmarsh, and Seaside Sparrows in North Carolina. Condor. 2012. 114:421–429.

[16] Sandercock BK, Jaramillo A. Annual survival rates of wintering sparrows: Assessing demographic consequences of migration. Auk. 2022. 149:149–165.

[17] Rising JD, Beadle DD. A guide to the identification and natural history of the sparrows of the United States and Canada. Academic Press, New York, NY, USA. 1996.

[18] Higgins KF, Kirsch LM, Ball IM Jr. A cable-chain device for locating duck nests. J Wildl Manage. 1969. 33:1009–1011.

[19] Petersen KL, Best LB. Nest-site selection by sage sparrows. 1985. Condor 87:217–221.

[20] Johnson RG, Temple SA. Nest predation and brood parasitism of tallgrass prairie birds. J Wildl Manage. 1990. 54:106–111.

[21] Kershner EL, Bollinger EK. Reproductive success of grassland birds at East-central Illinois airports. Am Midl Nat. 1996. 136:358–366.

[22] Trefry SA, Freedman B, Hudson JMG, Henry GHR. Breeding bird surveys at Alexandra Fiord, Ellesmere Island, Nunavut (1980–2008). Arctic. 2010. 63:308–314.

[23] McNair DB. Henslow’s sparrow and sedge wren response to a dormant-season prescribed burn in a pine savanna. Florida Field Naturalist. 1998. 26:46–47.

[24] Fletcher RJ, Dhundale JA, Dean TF. Estimating non-breeding season bird abundance in prairies: A comparison of two survey techniques. J Field Ornithol. 2010. 71:321–329.

[25] Heck BA, Arbour WD. The yellow rail in Oklahoma. Bul Oklahoma Ornithol Soc. 2008. 41:13–15.

[26] Shaw SM. Winter site fidelity in secretive marsh sparrows along the coast of South Carolina [dissertation]. Coastal Carolina University; 2012.

[27] Royle JA. Generalized estimators of avian abundance from count survey data. Anim Biodivers and Conserv. 2004. 27: 375–386.

[28] Chandler RB, Royle JA, King DI. Inference about density and temporary emigration in unmarked populations. Ecology. 2011. 92:1429–1435. doi: 10.1890/10-2433.1 21870617

[29] Sliwinski M, Powell L, Koper N, Giovanni M, Schacht W. Research design considerations to ensure detection of all species in an avian community. Methods Ecol Evol. 2016. 7:456–462.

[30] R Development Core Team. 2015. R: a language and environment for statistical computing. R Foundation for Statistical Computing, Vienna, Austria. [online]: URL: http://www.R-project.org

[31] Fiske I, Chandler R. Unmarked: an R package for fitting hierarchical models of wildlife occurrence and abundance. J Stat Softw. 2011. 43:1–23.

[32] Smith PGR. Observer and annual variation in winter bird population studies. Wilson Bull. 1984. 96:561–574.

[33] Best LG, Campa H, Kemp KE III, Robel RJ, Ryan MR, Savidge JA, et al. Avian abundance in CRP and crop fields during winter in the Midwest. Am Midl Nat. 1998 139:311–324.

[34] Weckerly FW. Constant proportionality in the female segment of a Roosevelt elk population. J Wildl Manage. 2007. 71(3): 773–777.

[35] Johnson DH. In defense of indices: the case of bird surveys. J Wildl Manage 2008. 72(40): 857–868.

[36] Bart J, Earnst S. Double sampling to estimate density and population trends in birds. Auk. 2002. 119:36–45.

[37] Cristol DA, Smith FM, Varian-Ramos CW, Watts BD. Mercury levels of Nelson’s and saltmarsh sparrows at wintering grounds in Virginia, USA. Ecotoxicology. 2011. 23:7. doi: 10.1007/s10646-011-0710-5 21698442

[38] Winder VL, Emslie SD. Mercury in breeding and wintering Nelson’s Sparrows (*Ammodramus nelsoni*). Ecotoxicology. 2011. 20:218–225. 21082242 10.1007/s10646-010-0573-1

[39] Eberhart-Phillips LJ, Küpper C, Miller TE, Cruz-López M, Maher KH, Dos Remedios N, et al. Sex-specific early survival drives adult sex ratio bias in snowy plovers and impacts mating system and population growth. Proceedings of the National Academy of Sciences. 2017. 114(27): E5474–E5481. doi: 10.1073/pnas.1620043114 28634289 PMC5502594

[40] Tolliver JD, Moore AA, Green MC, Weckerly FW. Coastal Texas black rail population states and survey effort. J Wildl Manage: 2019. 83(2): 312–324.

[41] Greenlaw JS, Rising JD. Sharp-tailed Sparrow (*Ammodramus caudacutus*). In The Birds of North America, No. 112 (Poole A. and Gill F., Eds.). Philadelphia: The Academy of Natural Sciences; Washington, D. C.; The American Ornithologists’ Union. 1994.

[42] Post W, Greenlaw JS. Nestling diets of coexisting salt marsh sparrows: Opportunism in a food-rich environment. Estuaries Coast. 2006. 29(5): 765–775.

[43] Sibley DA. Birds of Cape May. 2^nd^ ed. New Jersey Audubon Society, Cape May Bird Observatory, Cape May, NJ, USA; 1997.

[44] Gornitz VM, Daniels RC, White TW, Birdwell KR. The development of a coastal risk assessment database: Vulnerability to sea-level rise in the U.S. Southeast. J Coast Res. 1994: 12:327–338.

[45] Morris JT, Sunareshwar PV, Nietch CT, Kjerfve B, Cahoon DR. Response of coastal wetlands to rising sea-level. Ecology. 2002. 83:2869–2877.

[46] Craft C, Clough J, Ehman J, Joye S, Park R, Pennings S, et al. Forecasting the effects of accelerated sea-level rise on tidal marsh ecosystem services. Front Ecol Environ. 2008. 7:73–78.

[47] Chambers RM, Meyerson LA, Saltonstall K. Expansion of *Phragmites australis* into tidal wetlands of North America. Aquat Bot. 1999. 64:261–273.

[48] Kennish MJ. Coastal salt marsh systems in the U.S.: A review of anthropogenic impacts. J Coast Res. 2001 17:731–748.

[49] Deegan LA, Johnson DS, Warren RS, Peterson BJ, Fagherazzi S, Wollheim WM. Coastal eutrophication as a driver of salt marsh loss. Nature. 2012. 490:388–392. doi: 10.1038/nature11533 23075989

